# Comparative genomics of unintrogressed *Campylobacter coli* clades 2 and 3

**DOI:** 10.1186/1471-2164-15-129

**Published:** 2014-02-13

**Authors:** Caroline PA Skarp-de Haan, Alejandra Culebro, Thomas Schott, Joana Revez, Elke KH Schweda, Marja-Liisa Hänninen, Mirko Rossi

**Affiliations:** 1 Department of Food Hygiene and Environmental Health, Helsinki University, Helsinki, Finland; 2 Division of Chemistry, IFM, Linköping University, Linköping, Sweden

**Keywords:** *Campylobacter coli*, Comparative genomics, Phylogeny, Gamma glutamyltranspeptidase, Sialyltransferase

## Abstract

**Background:**

*Campylobacter jejuni* and *C. coli* share a multitude of risk factors associated with human gastrointestinal disease, yet their phylogeny differs significantly. *C. jejuni* is scattered into several lineages, with no apparent linkage, whereas *C. coli* clusters into three distinct phylogenetic groups (clades) of which clade 1 has shown extensive genome-wide introgression with *C. jejuni*, yet the other two clades (2 and 3) have less than 2% of *C. jejuni* ancestry. We characterized a *C. coli* strain (76339) with four novel multilocus sequence type alleles (ST-5088) and having the capability to express gamma-glutamyltranspeptidase (GGT); an accessory feature in *C. jejuni*. Our aim was to further characterize unintrogressed *C. coli* clades 2 and 3, using comparative genomics and with additional genome sequences available, to investigate the impact of horizontal gene transfer in shaping the accessory and core gene pools in unintrogressed *C. coli*.

**Results:**

Here, we present the first fully closed *C. coli* clade 3 genome (76339). The phylogenomic analysis of strain 76339, revealed that it belonged to clade 3 of unintrogressed *C. coli*. A more extensive respiratory metabolism among unintrogressed *C. coli* strains was found compared to introgressed *C. coli* (clade 1). We also identified other genes, such as serine proteases and an active sialyltransferase in the lipooligosaccharide locus, not present in *C. coli* clade 1 and we further propose a unique scenario for the evolution of *Campylobacter ggt*.

**Conclusions:**

We propose new insights into the evolution of the accessory genome of *C. coli* clade 3 and *C. jejuni*. Also, *in silico* analysis of the gene content revealed that *C. coli* clades 2 and 3 have genes associated with infection, suggesting they are a potent human pathogen, and may currently be underreported in human infections due to niche separation.

## Background

*Campylobacter jejuni* and *C. coli* are the most common bacterial cause for gastroenteritis in industrialized countries [1] and have been implicated in the development of several post-infectious sequelae [2,3]. Most research has focused on *C. jejuni*, but the role of *C. coli* in human disease is being increasingly recognized [4-7]. Both species share common risk factors for human infections, such as consumption of poultry, foreign travel, and drinking untreated water [6,8-10]. However, several case-case studies have also observed differences in the risk factors associated with either species, such as *C. coli* being more common in the elderly and those living in rural areas [4,7,9,11]. In addition, *C. jejuni* is most commonly found in poultry and ruminants, whereas *C. coli* colonizes pigs more frequently. Nevertheless, *C. coli* is also found in poultry, and it has been suggested that the populations circulating in these animal species are different [12].

Phylogenetic analyses by Sheppard et al. [13,14] have shown that *C. coli* strains cluster into three distinct phylogenetic groups (clades). In their analyses, both *C. coli* multilocus sequence type (MLST) ST-828 and ST-1150 clonal complexes were found in a clade (designated as introgressed clade 1) which showed extensive genome-wide introgression with *C. jejuni*[13,14]. On the contrary, many uncommon *C. coli* STs, not assigned to a clonal complex, clustered into two separate clades (unintrogressed clades 2 and 3) and showed less than 2% of *C. jejuni* ancestry, indicating that cross-species exchange had little or no impact on the gene pools of these lineages [14].

Although the ST-828 clonal complex accounts for the majority of *C. coli* infections in Finland, we recently identified a *C. coli* isolate (76339) from a patient with a domestically acquired infection which had a novel multilocus sequence type (ST-5088) and was deposited into the PubMLST database (http://pubmlst.org/campylobacter/) [15]. Further characterization of this strain showed that it produced gamma-glutamyltranspeptidase (GGT), which belongs to the accessory genome of *C. jejuni*. GGT is widely distributed in living organisms and is highly conserved. It belongs to the core genome of all gastric *Helicobacter* species and some enterohepatic *Helicobacter* spp. [16]. However, among *Campylobacter* spp. it has been detected in only a subset of *C. jejuni* strains [17], and has shown a strong association with only certain *C. jejuni* STs [18,19]. The presence of GGT in *C. coli* has not been described before and opens a question concerning the real impact of cross-species gene exchange between *C. jejuni* and unintrogressed *C. coli* lineages.

To investigate the impact of horizontal gene transfer in shaping accessory and core gene pools in unintrogressed *C. coli*, we carried out an extensive genomic characterization of *C. coli*, with special emphasis on *C. coli* clades 2 and 3. We provide the first closed genome of a *C. coli* belonging to clade 3 (strain 76339) on which we have performed an in-depth analysis of the gene content and phylogeny. We further defined the core and pan genome of unintrogressed *C. coli* clades 2 and 3 [13,14] and propose a novel view on the evolution of these lineages and their accessory gene content. Finally, we show evidence for a sialylated lipooligosaccharide (LOS) locus structure; a novel feature for unintrogressed *C. coli* clade 3.

## Methods

### Bacterial strain 76339, DNA extraction and MLST

*C. coli* strain 76339 was isolated from a human patient with a domestically acquired infection in July 2006 [20]. The species was confirmed using species-specific PCR [21] and frozen at -70°C in skim milk with 20% glycerol. Subsequent cultivations were routinely done on Nutrient Agar (Oxoid, Basingstoke, England) supplemented with 5% horse/bovine blood.

DNA was isolated with the Wizard Genomic DNA Purification Kit (Promega, Mannheim, Germany). MLST was performed as described previously [22-25].

### Whole genome sequences and annotation

The genome of *C. coli* 76339 was obtained using 454 Titanium (Roche; performed by LGC Genomics GmbH, Berlin, Germany) with a > 30× fold coverage. A combination of a pair-end and 8 kb mate-pair libraries was assembled into a scaffold representing a circular chromosome using MIRA 3.2. [26], SSAKE [27], and the Staden software package [28]. Verification of the scaffold was performed using PCR and Sanger sequencing. The shot-gun sequences of 63 other *C. coli* strains (Additional file 1: Table S1) were either downloaded from the NCBI ftp server or kindly provided by Dr. Samuel Sheppard (College of Medicine, Swansea University). Of these 63 *C. coli* strains, 54 were belonging to clade 1, four to clade 2 and five to clade 3. For gene finding and automatic annotation, the complete genome sequence of *C. coli* 76339 and all the other *C. coli* shot-gun sequences were uploaded to the RAST server [29]. The coding sequence of *C. coli* 76339 was further analysed using the Artemis tool [30] and manually re-annotated the genes of special interest. In particular, homology was identified using NCBI’s BLAST suite of programs with UniProtKB/Swiss-Prot as reference database and the conserved functional domains in proteins were identified using InterProScan [31]. For the prediction of glycosyltransferases we referred to the annotation available in the CAZy database [32]. The genomes of *C. coli* and *C. jejuni* used in this study are listed in Additional file 1: Table S1.

### Phylogenetic analysis

For the phylogenomics of *C. coli* and *C. jejuni,* the downloaded genomes were aligned with the multiple whole genome alignment tool Mugsy [33] by using the “-distance 1000” and “-minlength 100” options, as previously described [34]. The MAF blocks were concatenated and transformed in FASTA file format using the script available in Galaxy [35-37]. The resulting core alignment was filtered using Gblocks [38] with the minimum length of a block set at 100 (b4 = 100). A maximum likelihood tree was built using FastTree 2, applying the generalized time-reversible model (GTR) [39,40]. The model of evolution was selected using jmodeltest 2 [41]. In order to reconstruct the species tree of *C. coli*, a second analysis was performed. A fraction of the core genomes (calculated with OrthoMCL, see below) of *C. coli* strains 317_04, RM2882, BIGS10 and 76339 (each representing one of the four major monophyletic phylogenetic groups) which showed orthologs in the outgroup species *C. upsaliensis* was selected. Alignments for each of the one-to-one rooted core genes (543 orthologs) were first generated at the amino acid level using MAFFT-FFT-NS-i v.7 [42], then back-translated to nucleotide sequence using Translatorx perl script [43]. To account for the presence of possible recombination between the strains, each gene alignment was analysed using 3Seq in fullrun mode, setting the Bonferroni-corrected P-value cut-off at 0.05 [44,45], and using Pairwise Homoplasy Index [46], Maximum *χ*^2^[47] and the Neighbour Similarity Score [48], all implemented in PhiPack package [49]. The programme PhiPack was run by setting window size at 5 and the p-value of observing the sequences under the null hypothesis of no recombination at 0.05. To assess significance, 100 permutations tests were performed. Genes identified as unrecombined by all the four methods were selected for further analysis. The phylogenetic trees of each aligned unrecombined gene and of the concatenated alignments were inferred using PhyML [50] by applying the following parameters: -b -2, -m GTR, -o tlr, -a e, -c 6. A consensus tree based on the 543 maximum likelihood trees was generated using the extended Majority Rule method implemented in CONSENSE program available in PHYLIP package [51].

The phylogenetic trees of gamma-glutamyl transpeptidases (GGTs), sialyltransferases (Cst) and 16S rRNA genes were reconstructed using Bayesian phylogenetic inference. Homologs of GGT and Cst sequences were available from previous studies [16,52]. The nucleotide sequences were aligned based on amino acid alignment using PRANK by applying the TranslatorX perl script [43]. A multisequence alignment of full-length 16S rRNA genes of the type bacterial strains belonging to ϵ-proteobacteria was downloaded from the RDP website [53]. Two independent analyses of four MCMC chains run for 10 million generations with a tree sample each 10,000 generations were conducted for each gene using MrBayes v 3.2.1 [54]. GTR (nucmodel = 4by4 nst = 6) was selected as evolutionary model and the number of discrete categories used to approximate the gamma distribution was set to 6 (rates = gamma ngammacat = 6). To determine whether the data sets support conflicting phylogenies or a single tree, Neighbor-net networks were generated using Splitstree 4 [55].

### Comparative genomics

Orthologous and paralogous groups were determined using OrthoMCL version 2.0.2 [56]. A database of 111,061 amino acid sequences, including all the translated coding sequences (tCDSs) of annotated 64 *C. coli* genomes, was assembled (Ccoli-DB). Reciprocal all-versus-all BLASTP was performed and the results were processed by OrthoMCL using default parameters (thresholds to blast result: E-value < 1e-5, percent match length ≥ 50%) [56]. The OrthoMCL output was filtered to produce different lists of ortholog/paralog groups which contained: (i) tCDSs from all *C. coli* strains (core genome); (ii) tCDS from all the genomes of a clade; (iii) tCDSs from all the genomes of a clade and missing in the other clades; (iv) tCDSs from at least one genome of a clade and missing in the other clades.

To identify common orthologs between *C. coli* 76339 and the other 63 *C. coli* strains (Additional file 1: Table S1), a second approach was used. The complete set of predicted proteins of *C. coli* 76339 was compared to the pan-proteome including *C. coli* strains belonging to clade 1, clade 2 or clade 3, by reciprocal BLASTP using BLAST score ratio (BSR). The BSR was computed as previously described [57]. For each dataset, the BLAST raw score for each *C. coli* tCDS against itself was stored as the Reference score. Each *C. coli* tCDS was then compared to each tCDS of the *C. coli* 76339 predicted proteome with each best BLAST raw score recorded as Query score. The BSR is calculated by dividing the Query score by the Reference score for each tCDS. A cut-off of 0.4 was used to define if two tCDSs were homologs. This approach is more stringent than OrthoMCL and able to separate distant proteins which may be clustered in the same group by MCL.

### Phenotypic analysis

GGT activity was measured qualitatively as described before [58]. The LPS was extracted from *C. coli* 76339 grown in Nutrient Broth for 24 hours using the hot phenol-water method, and subjected to high performance anion-exchange chromatography with pulsed amperometric detection (HPAEC-PAD) for the detection of sialic acid, as previously described [52].

## Results and discussion

### General features of *C. coli* 76339 and definition of the core genome

**Figure 1:**
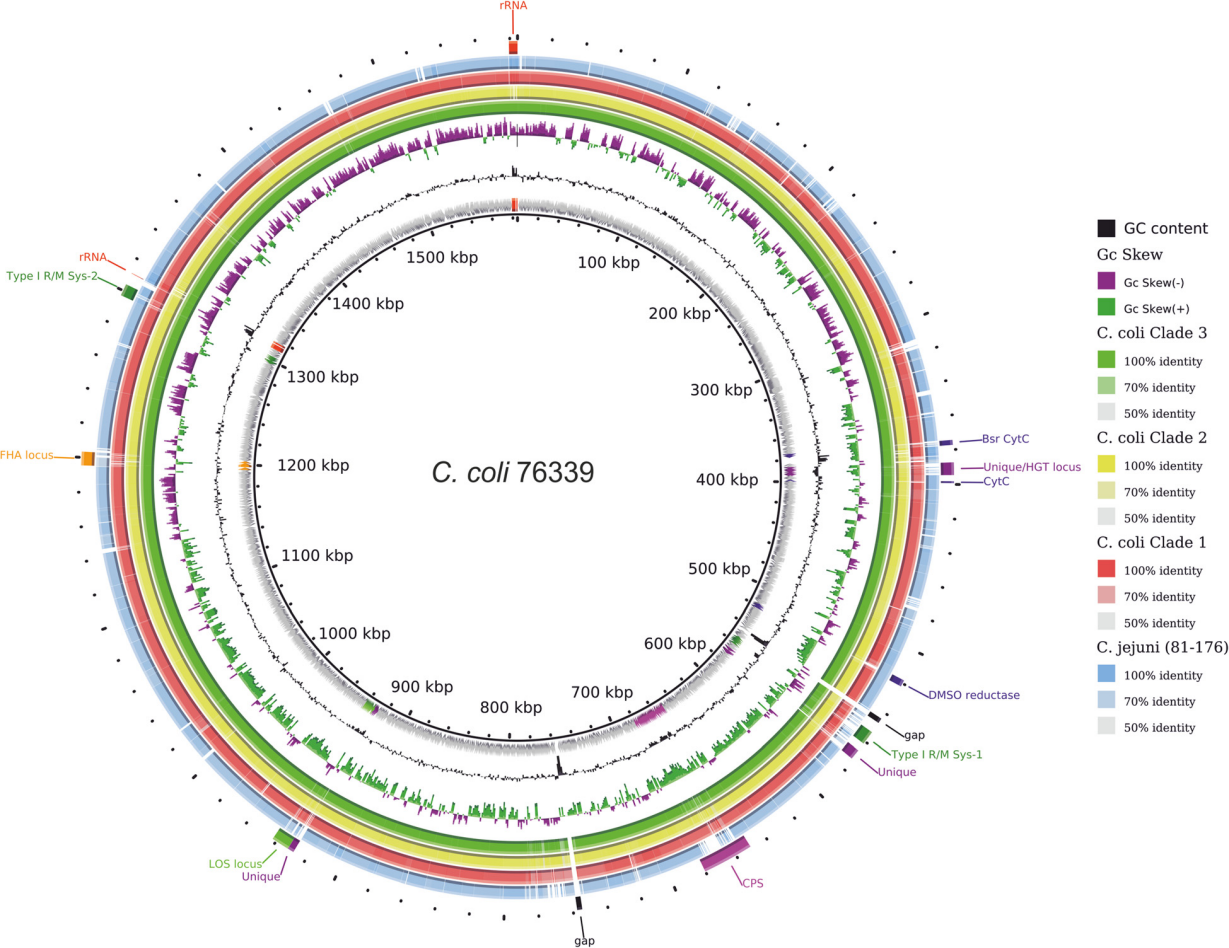
**Circular plot of the *C. coli *76339 chromosome, with highlighted features rRNA, biotin sulfide reductase (bsr), cytochrome C (CytC), DMSO reductase, Type I restriction modification systems, lipooligosaccharide locus (LOS) and filamentous haemagglutinin domain protein (FHA) locus.** GC content (black circles) and GC skew (green and purple circles) are represented. *C. jejuni* 81*–*176 and the pangenomes of each *C. coli* clade were compared.

A summary of the features of *C. coli* 76339 is given in Table 1 and a circular plot of the chromosome is presented in Figure 1. The genome of *C. coli* 76339 consists of a single chromosome which includes 1,556 protein-coding sequences (CDSs) in a coding area of 93.4%. A putative function could be predicted for 1,412 (90.7%) of the CDSs, whereas 144 (9.2%) of the CDSs were annotated as hypothetical proteins. Plasmids, insertion sequences (IS), prophages, and genomic islands were not detected in *C. coli* 76339, differentiating this strain from *C. coli* RM2228 [59]. Compared to other published *C. coli* and *C. jejuni* genomes, 76339 strain has a smaller chromosome and, consequently, possesses a lower number of CDSs [59,60].

**Table 1:** **Features of the *Campylobacter coli *** **76339 genome**

**Feature**	**Strain**	
	76339	RM2228
Origin	Clinical (stool)	Chicken
Multilocus sequence type	ST-5088	ST-1063
Clonal complex	-	828
Allelic profile (*aspA:glnA:gltA:glyA:pgm:tkt:uncA*)	121:278:328:431:552:452:154	33:39:30:140:113:43:41
*C. coli* clade	3	1b
Chromosome size	1,584,486 bp	~ 1.68 Mb
GC content	32.26%	31.37%
Coding sequences (CDS)	1556	1764
Assigned function	1412	1304
Hypothetical proteins	144	336
Restriction/Modification systems		
Type I	2	1
Type II	1	2
CRISPR	Yes	No

Based on MCL clustering, 97% of 111,061 the *C. coli* translated CDSs (tCDSs) included in the analysis could be divided into 2,951 groups of orthologs (GOs). A total of 1,524 GOs were detected in the proteome of *C. coli* 76339 comprising 98.8% of the complete set of tCDSs. Only 18 tCDSs did not belong to any GOs, and were unique for *C. coli* 76339 (Table 2). The core genome of *C. coli*, defined as the list of orthologs present in all *C. coli* strains, consisted of 654 GOs. This value was lower than that found with a previous OrthoMCL analysis performed with 42 *C. coli* strains [60]. In the study of Lefébure et al. [60], the core genome of *C. coli* was defined differently allowing a single strain to miss a core gene, and the authors estimated the core genome to include 1,485 GOs. However, even with a more relaxed definition, allowing a single strain to miss multiple core genes, we estimated the core genome of *C. coli* to have only 891 GOs. Our estimation is quite similar to the size of the core genome of the genus *Campylobacter* which comprises 647 OrthoMCL GOs [61].

**Table 2:** **Unique CDSs of *C. coli *** **76339**

**Locus tag**	**RAST annotation**
BN865_00400c	GTP-binding protein EngA
BN865_01640	Methyl-accepting chemotaxis signal transduction protein
BN865_01690	Diacylglycerol kinase (EC 2.7.1.107)
BN865_01750	Highly acidic protein
BN865_02020c	DNA modification methylase (Adenine-specific methyltransferase) (EC 2.1.1.72)
BN865_03900	Putative periplasmic protein
BN865_04140	Hypothetical protein
BN865_04150	Hypothetical protein
BN865_04190	Hypothetical protein
BN865_06070	Filamentous haemagglutinin domain protein
BN865_09290	Membrane protein
BN865_09810c	Hypothetical protein
BN865_09820c	Probable poly(beta-D-mannuronate) O-acetylase (EC 2.3.1.-)
BN865_10710	Small hydrophobic protein
BN865_11290	Putative mechanosensitive ion channel
BN865_12980c	Predicted permease YjgP/YjgQ family
BN865_13630c	ABC transporter, ATP-binding protein-related protein
BN865_13830	Putative integral membrane zinc-metalloprotease

### Phylogenomics of *C. coli* 76339 and delineation of *C. coli* species tree

**Figure 2:**
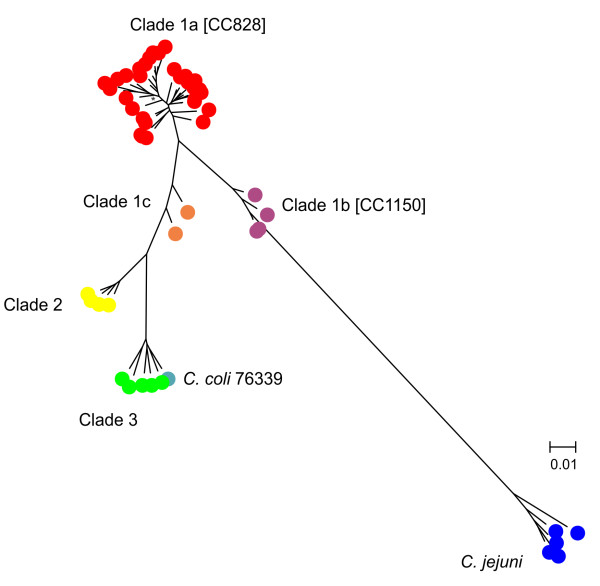
**Phylogram representing maximum-likelihood tree of *C. jejuni *and *C. coli*.** The phylogenetic position of *C. coli* 76339 is shown. All nodes were supported >80%, except for one node which was supported 51% and is indicated with an asterisk.

The phylogenetic position of *C. coli* 76339 is shown in Figure 2. The whole-genome alignment of 4,772,631 bp, including 64*C. coli* genomes and five *C. jejuni* strains (Additional file 1: Table S1), were treated with Gblocks which resulted in a gap-less multi-sequence alignment of 347,477 bp (~7% of the original multi-sequence alignment), which was used to build a Maximum Likelihood (ML) tree. The topology of the ML tree, based on the whole-genome alignment, resembled the neighbour-joining tree based on average genetic distances previously published by Sheppard et al. [14], and placed *C. coli* 76339 in clade 3 of unintrogressed strains [14]. In both the ML and the NJ tree, the branch of *C. jejuni* intersects the *C. coli* tree between clade 1a (ST-828 CC) and 1b (ST-1150 CC). As previously described, this position does not reflect the true evolution of *C. coli*, but instead is a consequence of introgression (interspecies recombination) of clade 1, and in particular of *C. coli* CC 1150, with *C. jejuni*[14]. This result indicates that interspecies recombination influences the topology of a ML tree when based on whole-genome alignment.

**Figure 3:**
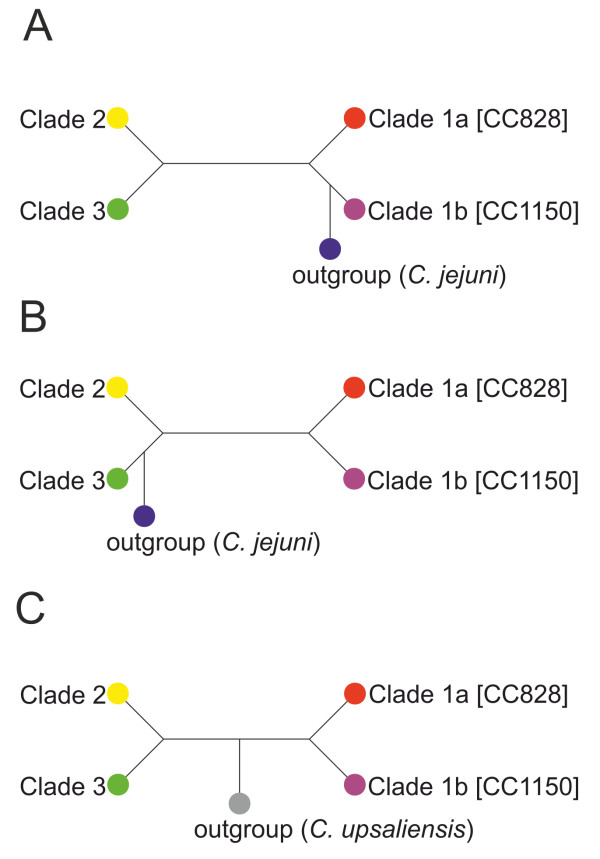
**Comparison of the topology of a *C. coli *phylogenetic tree obtained with different approaches. A**. Based on whole genome alignment using *C. jejuni* as an outgroup [13]. **B**. Based on 35 unrecombined *rps* genes using *C. jejuni* as an outgroup [14]. **C**. Based on 543 core genes using *C. upsaliensis* as an outgroup.

A previous study showed that in a tree based on 35 ribosomal proteins with no evidence of homologous recombination, the branch containing *C. jejuni* intersected the *C. coli* tree near to clade 3 [14]. Rooting this tree using *C. jejuni* as an outgroup showed that clade 3 has evolved from a common ancestor before the separation of clade 1 and 2 [Figure 3A and B, ref. [14], indicating that the unintrogressed *C. coli* strains are paraphyletic. In order to verify the evolution of *C. coli*, we inferred the species tree using a different approach. We selected one genome for each *C. coli* clade and *C. upsaliensis,* which has been demonstrated to be a sister group to the *C. jejuni*/*C. coli* clade [61], was chosen as an outgroup. We selected a total of 228 core genes out of 543 showing no statistically significant recombination among the strains. The ML tree obtained after concatenating those 228 unrecombined core genes showed that *C. upsaliensis* intersects the *C. coli* tree between clade 1, and clades 2 and 3 (Figure 3C). Both nodes are well supported with *χ*^2^-based parametric branch values of > 99%. In addition, the same topology was inferred by estimating the consensus tree of each of the 228 single gene trees using the extended majority rule method (data not shown), supporting the results obtained with concatenated genes. In fact, the splits ‘clade1a, clade1b | *C. upsaliensis,* clade 2, clade 3’ and ‘clade1a, clade1b, *C. upsaliensis* | clade 2, clade 3’ were present in 60.9% and 43.4% of the gene trees, respectively. In contrast, the split ‘clade 2, clade1a, clade1b | *C. upsaliensis,* clade 3’, which would support the topology of the concatenated unrecombined *rps* genes proposed by Sheppard et al. [14], was present in only 28% of the gene trees.

In summary, our data analysis showed that the unintrogressed *C. coli* strains are monophyletic (Figure 3C), suggesting a different evolutionary history than proposed by Sheppard et al. [14].

### Unique features of *C. coli* clade 3

A total of 1,282 GOs (84% of the GOs detected in *C. coli* 76339) were detected in all the studied strains belonging to clade 3. However, only six GOs were unique to this clade: a putative protease (GO-CCO3301; BN865_02000), a protein belonging to Cytochrome-c family (GO-CCO3300; BN865_04240) and a second DMSO reductase system (includes four GOs: chain A GO-CCO3235, BN865_05620; chain B GO-CCO3303, BN865_05610; chain C, GO-CCO3302, BN865_05600; chain D GO-CCO3392, BN865_05590 which was missing in one strain of clade 3).

### Serine proteases

The first GO unique to *C. coli* clade 3 strains includes a protease (BN865_02000) which was found to contain an immunoglobulin A1 protease domain in the N-terminus and an autotransporter in the C-terminus. In three strains of clade 3, the protein is probably fragmented and homology was found only in the C-terminal part. This protein belongs to the MEROPS peptidase family S6 and bears significant homology to members of the autotransporter family, such as the serine protease autotransporters of *Enterobacteriacae* (SPATE) [62]. It had significant BLASTP hits with putative uncharacterized serine proteases of *C. jejuni* (e.g. 47% amino acid identity with CJM1_0203 of *C. jejuni* M1). However, the homology is limited to the N-terminus of the sequence and does not include the autotransporter domain. Reciprocal BLASTP allowed the identification of another serine protease autotransporter in *C. coli* 76339 (BN865_07680) which gave a significant BSR (> 0.4) with sequences of only clade 3 strains. These sequences belong to the GO-CCO3075, which also contains four proteins present in *C. coli* clade 1 strains. These proteins share the same domains and belong to the MEROPS peptidase family S8A, which includes homologs to subtilisin [62]. The clade 3 subtilisin-like proteins were distantly related to homologs of *C. upsaliensis* (55% identity) and *C. jejuni* 81–176 (53% identity with CJJ81176_1371). In contrast, the clade 1 subtilisin-like protein was 100% identical to the *C. jejuni* 81–176 serine protease CJJ81176_1367. This indicates that the evolutionary dynamics of both clade 3 serine proteases is difficult to predict. The monophyletic relationship between clade 2 and 3 (Figure 3C) suggests gene extinction would not be parsimonious and thus horizontal gene transfer (HGT) could have played a major role. Nevertheless, gene extinction cannot be completely excluded and would be well supported by the topology of the species tree proposed by Sheppard et al. [14] in which clades 2 and 3 are paraphyletic.

### Cytochrome-c family protein and a second DMSO reductase system

Additional features that characterized *C. coli* clade 3 were the Cytochrome-c (CytC) family protein and a second DMSO reductase system. Both are likely involved in the respiratory chain, and may confer a metabolic advantage to these strains. Both systems have homologs in *C. jejuni*; CytC BN865_04240 showed 91% nucleotide identity with Cj0037 of *C. jejuni* NCTC 11168 and may have been exchanged between *C. jejuni* and *C. coli* clade 3. The second DMSO system is organized as described in *C. jejuni* 81–176 [63] and is located in the same region of the genome, yet it’s lower amino acid identity with *C. jejuni* 81*–*176 (~80% amino acid identity between BN865_05620 and CJJ81176_1570) suggests an origin different from CytC.

As observed for the serine proteases, a scenario of gene extinction would be supported by the topology of the species tree proposed by Sheppard et al. [14]. The monophyletic relationships between clade 2 and 3 that we found, however, suggests that *C. coli* clade 3 and certain lineages of *C. jejuni* might have acquired the second DMSO system by HGT from independent sources. This makes it tempting to speculate that during the evolution of *C. coli* clade 3 the second DMSO system might have been acquired as a consequence of niche adaptation.

### Additional features of *C. coli* clade 3

In addition to the six specific *C. coli* clade 3 GOs, a total of 18 extra GOs were found to be present in at least one genome of clade 3, but missing in the other *C. coli* genomes (Table 3). Several of these groups contain small putative proteins with unknown function, and only a few were also detected in *C. coli* 76339: a hypothetical protein containing a C-terminal autotransporter domain (BN865_03550); a hemerythrin family non-heme iron protein (BN865_01820) and two other hypothetical proteins (BN865_05590; BN865_10320).

**Table 3:** **Group of orthologs (GO) unique to *C. coli *** **clade 3**

**Group of orthologs (GOs)**	**RAST annotation**	**Locus tag 76339**
CCO3388	Nitroreductase family protein	
CCO3390	Hypothetical protein	
CCO3392	Hypothetical protein CJJ81176_1573	BN865_05590c
CCO3393	Hemerythrin family non-heme iron protein	BN865_01820
CCO3489	Hypothetical protein (autotransporter)	BN865_03550
CCO3490	Small putative protein	BN865_10320
CCO3520	NADPH:quinone reductase and related Zn-dependent oxidoreductases	
CCO3645	Small putative protein	
CCO3646	Major antigenic peptide PEB3	
CCO3648	Small putative protein	
CCO3649	Small putative protein	
CCO3793	Small putative protein	
CCO3794	Small putative protein	
CCO3799	Small putative protein	
CCO3800	Methyltransferase	
CCO3801	Small putative proteIn	
CCO3802	Small putative protein	
CCO3880	Small putative protein	

The GOs describe here were present in at least one genome of *C. coli* clade 3, but missing in all other *C. coli* strains.

### TonB2 and GGT are two common features characterizing unintrogressed clade 2 and 3 *C. coli* strains

A total of 25 GOs were detected to be common in *C. coli* strains belonging to clades 2 and 3 (present in at least one strain of both clades), but missing in clade 1 (Table 4). Among these, a gene homologous to *C. jejuni tonB2* (GO-CCO3049; BN865_05130) was found to be common in all the strains belonging to clades 2 and 3. In addition to *tonB2*, the gene encoding gamma glutamyltranspeptidase; *ggt* (GO-CCO3111; BN865_04090) was common in all but one unintrogressed *C. coli* strains.

**Table 4:** **Group of orthologs unique to *C. coli *** **clades 2 and 3**

**Group of orthologs (GOs)**	**RAST annotation**	**Locus tag 76339**
CCO3049	tonB2	BN865_05130
CCO3111	GGT	BN865_04090c
CCO3112	Small putative protein	
CCO3236	Small putative protein	BN865_04140
CCO3255	Type I R/M system	BN865_13650c
CCO3348	Small putative protein	
CCO3368	Small putative protein	
CCO3391	Type I R/M system	BN865_13620c
CCO3394	Chloramphenicol acetyltransferase	BN865_06010
CCO3395	Hypothetical protein	BN865_11580
CCO3433	DnaJ superfamily	
CCO3436	Small putative protein	
CCO3459	Hypothetical protein - *Helicobacter* homolog	BN865_10830
CCO3522	Small putative protein	
CCO3574	Aldo/keto reductase family	
CCO3575	Putative transcriptional regulator	
CCO3577	Small putative protein	
CCO3674	Type I R/M system	BN865_13640c
CCO3724	Aldo/keto reductase family	
CCO3726	Small putative protein	
CCO3737	Small putative protein	
CCO3896	Recombination protein T	
CCO3899	Small putative protein	
CCO3945	Small putative protein	
CCO3946	Small putative protein	

The GOs described here were present in at least one genome of *C. coli* clade 3 and 2, but missing in *C. coli* clade 1.

### TonB2 transport protein

The TonB protein is involved in iron acquisition and exists in a complex with ExbB and ExbD, which provides the energy for transport of ferric (Fe^3+^) iron through the outer membrane receptors [64-66]. So far, a total of three *tonB* homologs have been described in *C. jejuni* and the majority of *C. jejuni* strains contain all genes, yet some strains (e.g. *C. jejuni* 81–176 and 81116) possess only *tonB2*[67]. Similar to *C. jejuni*, *tonB1* and *tonB3* belong to the core genome of *C. coli* and due to the surplus of sequenced *C. coli* clade 1 strains, the presence of a third *tonB* gene in *C. coli* was unknown*.* Here, we show, for the first time, the presence of *tonB2* in *C. coli*, which is limited to clade 2 and 3 strains.

### Gamma glutamyltranspeptidase

**Figure 4:**
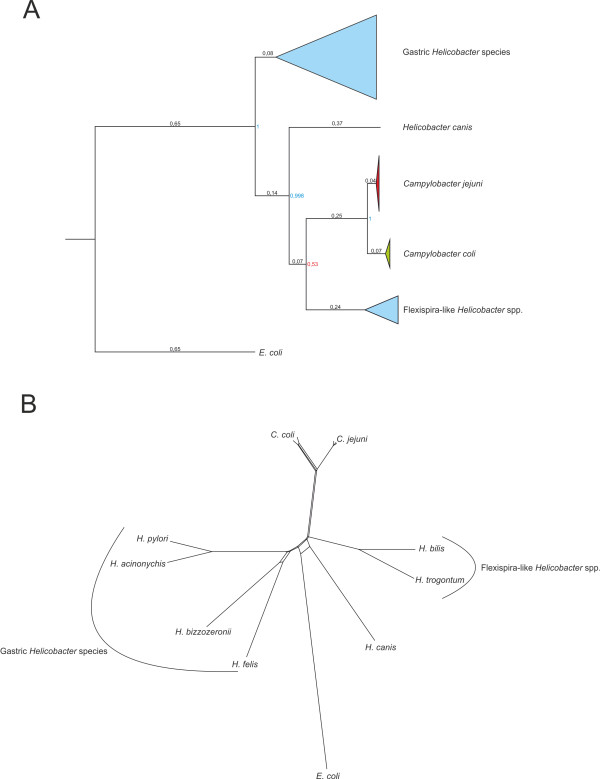
**Bayesian phylogeny and split decomposition of *ggt *orthologs in ϵ-proteobacteria. A**. Bayesian phylogeny of *ggt* orthologs in different ϵ-proteobacterial species, rooted with the *ggt* sequence of gamma-proteobacterium *Escherichia coli* (*E. coli*). From down to up: *Helicobacter bilis* and *H. trogontum* (represented as Flexispira-like *Helicobacter* spp.); *Campylobacter coli*; *C. jejuni*; *H. canis* and *H. pylori*, *H. acinonychis*, *H. bizzozeronii* and *H. felis* (represented as Gastric *Helicobacter* species). Numbers on the branch indicate distance values and the numbers on the nodes indicate posterior probability. Posterior probability values indicated in blue font are >70% and those indicated in red font are <70%. **B**. Split decomposition analysis of *ggt* orthologs in *C. jejuni* and *C. coli* and *Helicobacter* species. Absence of a netlike structure between *C. jejuni* and *C. coli* indicates absence of HGT between the two species.

Similar to *C. jejuni*, the *ggt* gene in *C. coli* 76339 is located downstream of a ribosomal operon, which is considered to be a recombinational hotspot and together with the accessory nature of *C. jejuni ggt*[17], this suggests that the *C. coli ggt* could have been acquired by HGT [68]. In order to investigate the possible origin of *ggt* in *Campylobacter* spp., the phylogeny of *ggt* orthologs in ϵ-proteobacteria was reconstructed using Bayesian inference (Figure 4A) and compared to a Bayesian species tree of the ϵ-proteobacteria based on the small ribosomal unit (Additional file 2: Figure S1). Both tree topologies support the hypothesis that *ggt* was acquired by an ancestral *Campylobacter* species through HGT and originated from an ancestral *Helicobacter* species. However, after acquisition and during evolution of both *C. jejuni* and *C. coli*, the gene underwent progressive extinction. This hypothesis is supported by several lines of evidence. First, the presence of *ggt* in only unintrogressed *C. coli* isolates suggests that the gene evolved separately after speciation and was not exchanged between the two species. This is corroborated by a split decomposition analysis which showed no net-like structure between *C. coli* and *C. jejuni ggt* (Figure 4b). Furthermore, the gene extinction scenario is also supported by the topology of both proposed *C. coli* species trees (Sheppard et al. [14] and this study). Progressive extinction could also be inferred for *C. jejuni*. The rooted ML tree, representing the evolution of *C. jejuni* (Additional file 3: Figure S2), shows that *ggt* gradually disappears while moving away from the root. In *C. jejuni*, *ggt* is typically found in multilocus sequence types (STs) that are predominant in chickens opposed to those STs that are predominant in bovines and barnacle geese [18]. Therefore, the original advantage associated with the acquisition of *ggt* may have vanished during the adaptation of *C. coli* and *C. jejuni* as a consequence of niche segregation [69].

### Additional features of unintrogressed *C. coli* strains

*C. coli* 76339 possesses, in common with three other clade 3 strains and one clade 2 strain, a gene containing a chloramphenicol acetyltransferase domain (GO-CCO3394; BN865_06010) which is located immediately downstream of a highly conserved alcohol dehydrogenase (GO-CCO1275; BN865_06000). Although *C. coli* 76639 expressed BN865_06010 in vitro, the MIC for chloramphenicol was lower than 1 mg/L (data not shown), indicating that the gene may not be able to confer resistance to chloramphenicol and is probably misannotated.

Another interesting feature is the structure known as clustered regularly interspaced short palindromic repeat (CRISPR) locus, which is considered to function as a prokaryotic immune system and protects against invasion of alien genetic elements, e.g. plasmids and phages [70]. The CRISPR locus of *C. coli* clades 2 and 3 consists of four spacers and a putative trans-encoded sRNA sequence (based on nucleotide similarity with *C. jejuni* 81116 tracrRNA [71]). The CRISPR/cas system in *C. coli* 76339 and other clade 3 strains possess only the *cas9* gene (GO CCO2663; BN865_15240c), but homologs for *cas1* and *cas2* are absent. Homologs of the *C. jejuni* CRISPR/cas system were found in all strains belonging to clade 2 and a subset of clade 1 strains. However, the location of the CRISPR/cas system in the genomes distinguishes introgressed clade 1 from unintrogressed *C. coli* clades 2 and 3. In unintrogressed *C. coli* clades 2 and 3 the CRISPR locus is found between *rodA* and *dnaB,* whereas in the strains of clade 1 the locus is located in the same position of the genome as described for *C. jejuni* (between *moeA2* and *purM*[71]). These data corroborate the hypothesis of interspecies recombination between *C. coli* clade 1 and *C. jejuni* proposed by Sheppard et al. [14] as well as the monophyletic relationship between *C. coli* clade 2 and 3.

### Gene flow between *C. coli* clades

Sheppard et al. [14] estimated a 4% genetic exchange between the three *C. coli* clades. We found several genes in our *C. coli* 76339 which were absent in other clade 3 strains, but present in clade 1 or 2 (Table 5). A high Blast score ratio was found for half of the capsule polysaccharide (CPS) locus genes with *C. coli* clade 1a (ST-1150 CC) [72]. These genes were absent in the other *C. coli* clade 1 and 2 strains. In addition, several genes encoding methyl-accepting chemotaxis signal transduction proteins were found; all of which were also present in *C. coli* clade 1a, but not always in clades 1b, 1c and 2. Finally an oxygen-insensitive NAD(P)H nitroreductase was commonly found among clades 1 and 2 and our *C. coli* 76339, but absent in other clade 3 strains. Thus, gene flow among *C. coli* clades is possible and probably depends on a number of factors facilitating homologous recombination, such as a shared ecological niche or transient co-colonization of the same host.

**Table 5:** **Genes possibly acquired through gene flow by *C. coli *** **76339**

**Locus tag**	**RAST annotation**	**Present in**	**Missing in**
BN865_01630	Methyl-accepting chemotaxis signal transduction protein	C1a; C1b	C1c; C2
BN865_02140	Methyl-accepting chemotaxis signal transduction protein	C1a; C2	C1b; C1c
BN865_02630c	Methyl-accepting chemotaxis signal transduction protein	C1a	C1b; C1c; C2
BN865_02680c	McrBC restriction endonuclease system, McrB subunit, putative	C2	C1a; C1b; C1c
BN865_03910	Conserved hypothetical secreted protein	C2	C1a; C1b; C1c
BN865_04310c	FIG 00470070: hypothetical protein	C1a	C1b; C1c; C2
BN865_05840c	Methyl-accepting chemotaxis signal transduction protein, fragment	C1a	C1b; C1c; C2
BN865_05850c	Methyl-accepting chemotaxis signal transduction protein, fragment	C1a	C1b; C1c; C2
BN865_06160	Hypothetical protein	C1a; C2	C1b; C1c
BN865_06170	Hypothetical protein	C1a; C2	C1b; C1c
BN865_06850c	Beta-1,3-glucosyltransferase	C1a; C2	C1b; C1c
BN865_07080	Hypothetical protein	C1a	C1b; C1c; C2
BN865_07090	2-dehydro-3-deoxyglucarate aldolase (EC 4.1.2.20)	C1a	C1b; C1c; C2
BN865_07100	D-3-phosphoglycerate dehydrogenase (EC 1.1.1.95)	C1a	C1b; C1c; C2
BN865_07110	NAD-dependent epimerase/dehydratase	C1a	C1b; C1c; C2
BN865_07120	Putative cyclase superfamily	C1a	C1b; C1c; C2
BN865_07130	Conserved hypothetical protein 22	C1a	C1b; C1c; C2
BN865_07140	Hydrolase, haloacid dehalogenase-like family	C1a	C1b; C1c; C2
BN865_07150	CMP-N-acetylneuraminate-beta-galactosamide- alpha-2,3-sialyltransferase (EC 2.4.99.-)	C1a	C1b; C1c; C2
BN865_07160	FIG 00470714: hypothetical protein	C1a	C1b; C1c; C2
BN865_07180	Haloacid dehalogenase-like hydrolase domain/phosphoribulokinase domain protein	C1a	C1b; C1c; C2
BN865_07190	Capsular polysaccharide biosynthesis protein, putative	C1a	C1b; C1c; C2
BN865_07200	Capsular polysaccharide biosynthesis protein, putative	C1a	C1b; C1c; C2
BN865_08140c	Methionyl-tRNA formyltransferase (EC 2.1.2.9)	C1a	C1b; C1c; C2
BN865_10680c	Oxygen-insensitive NAD(P)H nitroreductase (EC 1.-.-.-)/Dihydropteridine reductase (EC 1.5.1.34)	C1a; C1b; C1c; C2	-
BN865_10830	Hypothetical protein	C2	C1a; C1b; C1c
BN865_14240	Possible sugar transferase	C1a	C1b; C1c; C2
BN865_14270	Methyltransferase (EC 2.1.1.-), possibly involved in O-methyl phosphoramidate capsule modification	C1a; C1b	C1c; C2
BN865_14740	Family of unknown function (DUF450) family	C1a	C1b; C1c; C2

The genes listed here were absent in other clade 3 strains, but present in *C. coli* clades 1 or 2.

### Evidence of LOS sialylation of *C. coli* 76339

**Figure 5:**
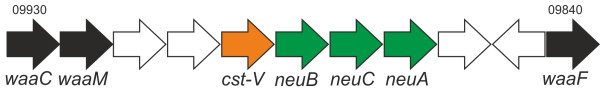
**Lipooligosaccharide (LOS) locus *C. coli *strain 76339 located between *waaC *(BN865_09930) and *waaF *(BN865_09840).** Conserved genes are indicated using black arrows. Putative glycosyltransferases are shown in white, sialyltransferase *cst-V* in orange and the sialic acid biosynthesis genes (*neuBCA*) in green.

Using a BLAST score ratio cut off of 0.4, a putative sialyltransferase (BN865_09900) was detected (Additional file 4: Table S2). This protein is located in the LOS locus upstream of three genes necessary for the biosynthesis and transfer of sialic acid (*neuABC*), resembling *C. jejuni* LOS locus classes A and B (Figure 5) [73] but not other *C. coli* LOS locus classes described by Richards and colleagues [72]. The presence of these particular genes in the LOS locus suggests that strain 76339 may express sialylated LOS structures [74]. HPAEC-PAD analysis of the purified LOS obtained from *C. coli* 76339 revealed the presence of sialic acid, supporting the genomic results. This finding is important because it would imply that certain *C. coli* could also have bacterial factors considered important in the pathogenesis of Guillain-Barré syndrome [2,3]. It remains unknown, however, onto which substrate the sialic acid is transferred and thus whether or not this structure would mimic human gangliosides, and further studies are needed to deduce the structure.

**Figure 6:**
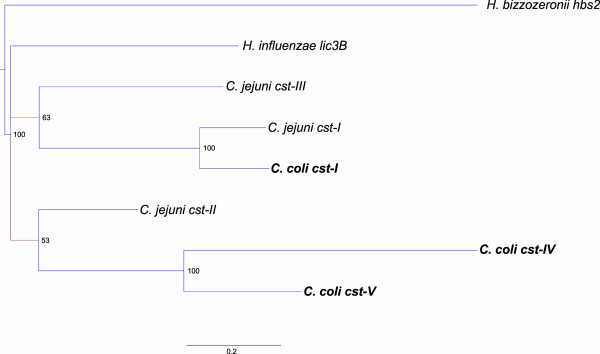
**Bayesian phylogeny of GT42 sialyltransferases indicating relatedness of *C. jejuni *and *C. coli *sialyltransferases.***C. coli cst-I* is found in the capsule (CPS) locus of *C. coli* clade 3 strains and is an ortholog of *C. jejuni cst-I. C. coli cst-IV* is found in the lipooligosaccharide (LOS) locus of *C. coli* clade 1 strains and *C. coli cst-V* is found in the LOS locus of *C. coli* clade 3 strains.

No evidence has been found of the presence of the *neuABC* gene cluster in the LOS locus of any of the 42 *C. coli* strains analyzed in a previous study [72], although it was evident in the CPS locus classes VII and VIII [72]. However, the authors found the presence of a putative sialyltransferase (named 1501) in two LOS classes of *C. coli* (class B and C). In our MCL cluster analysis we found that the putative *C. coli* 76339 sialyltransferase BN865_09900 belongs to GO-CCO2667 which includes several other sequences from both *C. coli* clade 1 and 3. All the sequences of GO-CCO2667, showed a significant homology to those belonging to the CAZy glycosyltransferase family GT42, supporting the idea that all encode putative sialyltransferases [32]. Further analysis revealed that the clade 1 GO-CCO2667 sequences corresponded to sialyltransferase 1501 identified by Richards et al. [72]. Additionally, *C. coli* 76339 possesses a second sialyltransferase (BN865_06990), which was found to be located in the CPS locus of the strain and to have an ortholog in other clade 3 strains. This protein gave no significant BLASTP hits with other *C. coli* sequences (BSR cut off 0.4), but it showed 67% identity with *C. jejuni* ATCC 43456 Cst-I. To elucidate the phylogenetic relationship among *Campylobacter* sialyltransferases we inferred the phylogeny of GT42 sequences by applying Bayesian methodology (Figure 6). The LOS-associated *C. coli* sialyltranferases were shown to be monophyletic and distantly related to *C. jejuni* sialyltransferases. We propose to name these genes Cst-IV (clade 1) and Cst-V (clade 3). The distant relationship observed between LOS-associated *C. coli* and *C. jejuni* sialyltransferases could indicate evolution of different substrate specificity, which has been previously observed among *Helicobacter* sialyltransferases [52]. As a consequence, these bacteria may express different sialylated structures on their LOS. On the contrary, the *C. coli* clade 3 sialyltransferases located within the capsule locus clustered tightly together with *C. jejuni* Cst-I, which supports the notion of interspecies HGT and the potential of sharing similar sialylated glycan structures on the surface.

## Conclusions

From a phylogenetic point of view we found *C. coli* clades 2 and 3 to be monophyletic, rather than paraphyletic [14], implying common ancestry, in which both gene extinction and HGT could play a plausible role in the separation of two distinct clades. Furthermore, unintrogressed *C. coli* clade 3 strains show potential for an extensive respiratory metabolism; possibly reflecting their wide host range and adaptability to novel niches. Finally, we propose a new insight into the evolution of the accessory genome of both *C. coli* and *C. jejuni*, which should be exploited further with other dispensable genes.

### Availability of supporting data

The genome of C. coli 76339 was deposited in EMBL under accession number HG326877. Trees were submitted to Treebase and are available for download at http://purl.org/phylo/treebase/phylows/study/TB2:S15193. The Ccoli-DB and the groups of orthologs are available at the University of Helsinki for download at http://www.mv.helsinki.fi/mirossi/C.coli-DB/ or upon request to the author.

## Competing interests

The authors declare that they have no competing interests.

## Authors’ contributions

CPAdH participated in the genome assembly and analysis and in drafting the manuscript. AC carried out experimental work. TS performed the genome assembly and annotation. JR was involved in characterization of the LOS locus. EKHS has carried out the phenotypic characterization of the LOS structure. MLH conceived the idea. MR has carried out the phylogenetic analysis, the comparative genomic and in drafting the manuscript. All authors have been involved in drafting of the manuscript. All authors read and approve the manuscript.

## Supplementary Material

Additional file 1: Table S1 List of *C. coli* and *C. jejuni* genomes used in this study. Click here for file

Additional file 2: Figure S1 Bayesian ϵ-proteobacteria species tree based on the small ribosomal unit. Click here for file

Additional file 3: Figure S2 Maximum likelihood tree rooted with *C. coli* 76339, representing the evolution of *C. jejuni*. The ML tree is based on whole genome sequence alignment, with a node support of >90%. Presence or absence of the *ggt* gene is indicated. *C. jejuni* clonal complexes are separated into seven clades. Click here for file

Additional file 4: Table S2 CDSs of *C. coli* 76339 with no significant BLASTP result versus other *C. coli* proteins. A BLAST score ratio (BSR) cut-off 0.4 was used. Click here for file
