# Supplementary figures and images for: Comparative genomics of unintrogressed Campylobacter coli clades 2 and 3
Source: BMC Genomics. 2014 Feb 13;15:129. doi: 10.1186/1471-2164-15-129 (PMC3928612; doi:10.1186/1471-2164-15-129)

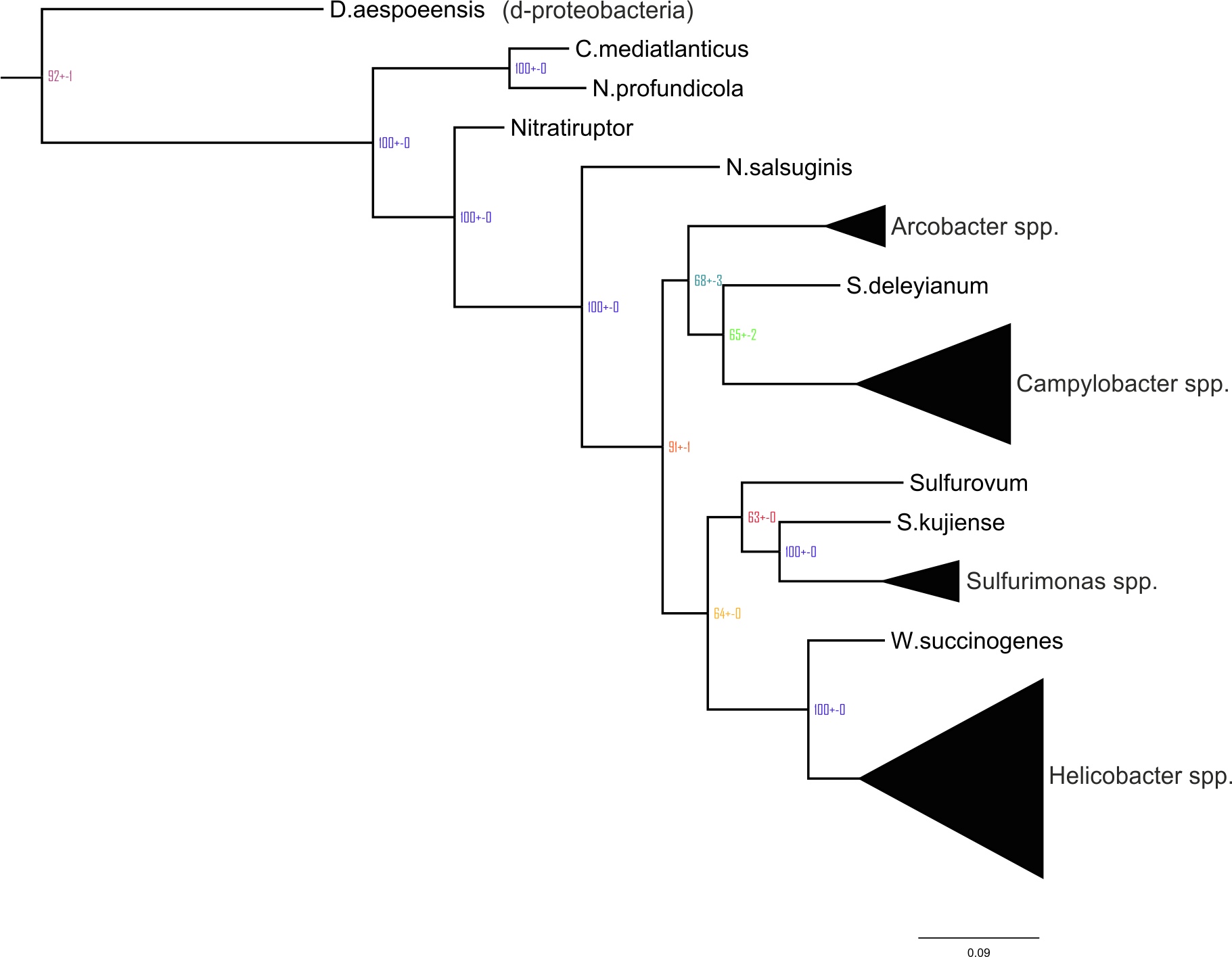

Supplement: Additional file 2: Figure S1 — Bayesian ϵ-proteobacteria species tree based on the small ribosomal unit. [file 1471-2164-15-129-S2.png]

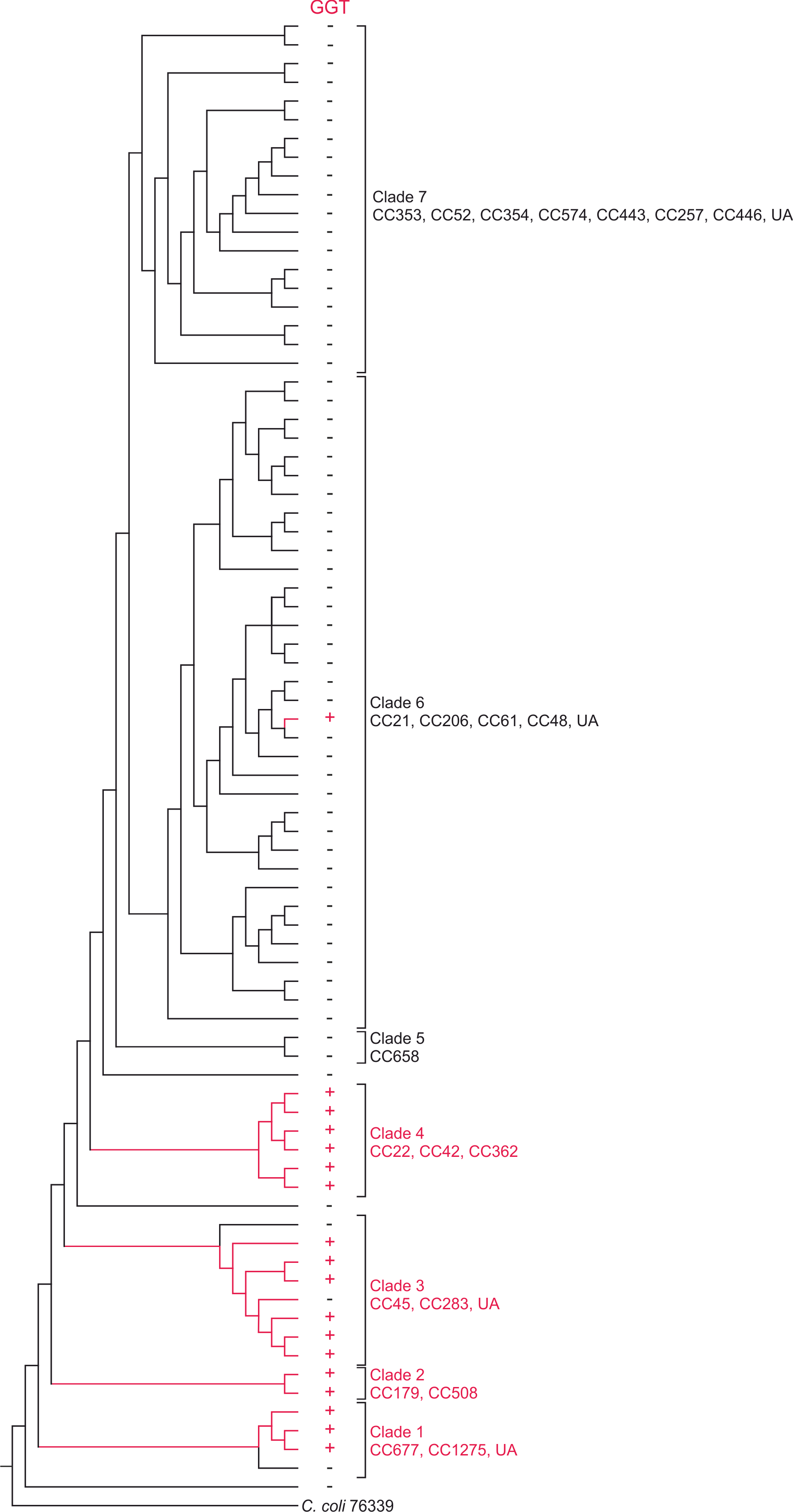

Supplement: Additional file 3: Figure S2 — Maximum likelihood tree rooted with C. coli 76339, representing the evolution of C. jejuni. The ML tree is based on whole genome sequence alignment, with a node support of >90%. Presence or absence of the ggt gene is indicated. C. jejuni clonal complexes are separated into seven clades. [file 1471-2164-15-129-S3.png]
