# Supplementary material for: Comparative genomics of unintrogressed Campylobacter coli clades 2 and 3
Source: BMC Genomics. 2014 Feb 13;15:129. doi: 10.1186/1471-2164-15-129 (PMC3928612; doi:10.1186/1471-2164-15-129)
Supplement: Additional file 4: Table S2 — CDSs of C. coli 76339 with no significant BLASTP result versus other C. coli proteins. A BLAST score ratio (BSR) cut-off 0.4 was used. [file 1471-2164-15-129-S4.docx]

| **Locus tag** | **RAST annotation** | **Best BLAST hit^1^** | **BLAST score ratio** |
| --- | --- | --- | --- |
| BN865_03900 | Putative periplasmic protein | Hypothetical protein cje133_04093 [*Campylobacter jejuni* subsp. *jejuni* LMG 23357] | 0,71 |
| BN865_04150 | hypothetical protein | No hit | - |
| BN865_04170 | Putative integral membrane protein | Hypothetical protein cje100_06782 [*Campylobacter jejuni* subsp. *jejuni* LMG 23216] | 0,40 |
| BN865_04180 | hypothetical protein | Hypothetical protein HMPREF9477_01701 [*Lachnospiraceae* bacterium 2_1_46FAA] | 0,28 |
| BN865_06060 | Putative periplasmic protein | Putative periplasmic protein [*Campylobacter jejuni* subsp. *jejuni* 1213] | 0,38 |
| BN865_07010 | Beta-1,3-galactosyltransferase /  Beta-1,4-galactosyltransferase | Wca [*Campylobacter jejuni* ATCC 43432] | 0,47 |
| BN865_07070 | FIG00855465: hypothetical protein | Hypothetical protein NB231_02758 [*Nitrococcus mobilis* Nb-231] | 0,30 |
| BN865_09790c | hypothetical protein | Hypothetical protein C8J_1094 [*Campylobacter jejuni* subsp. *jejuni* 81116] | 0,48 |
| BN865_09800c | unknown | Conserved hypothetical protein [*Campylobacter jejuni* subsp. *jejuni* 327] | 0,28 |
| BN865_09860c | general stress protein A, putative( EC:2.4.1.58 ) | Glycosyl transferase [*Helicobacter winghamensis* ATCC BAA-460] | 0,30 |
| BN865_09900c | CMP-N-acetylneuraminate-beta-galactosamide-alpha-2,3-sialyltransferase (EC 2.4.99.-) | Alpha-2,3/8-sialyltransferase [*Campylobacter jejuni*] | 0,37 |
| 0BN865_13640c | Type I restriction-modification system,  specificity subunit S (EC 3.1.21.3) | Hypothetical protein C414_000210006 [*Campylobacter jejuni* subsp. *jejuni* LMG 414] | 0,91 |

^1^E value < 0.001; Identity > 30%; query coverage > 60%
